# Design and operational features of medical alliances in China

**DOI:** 10.1038/s44401-026-00090-3

**Published:** 2026-05-12

**Authors:** Wu Zeng, Tianjiao Gao, Guanyang Zou, Guohong Li, Huihui Wang

**Affiliations:** 1 Department of Global Health, Georgetown University, Washington, DC USA; 2 Heller School for Social Policy & Management, Brandeis University, Waltham, MA USA; 3 Guangzhou University of Chinese Medicine, Guangzhou, China; 4 School of Global Health, Shanghai Jiaotong University School of Medicine, Shanghai, China; 5 World Bank, Washington, DC USA

**Keywords:** Development studies, Economics, Economics, Health humanities, Politics and international relations, Social policy

## Abstract

Many countries aim to promote patient-centered, effective, efficient, and collaborative health systems. This study provides an overview of key design and operational features of medical alliances in China. It highlights the critical role of governmental political will, investment in health financing systems, consolidation of health resources, development of appropriate incentives, and establishment of robust monitoring and evaluation mechanisms in facilitating the effective operation of medical alliances.

## Introduction

Many countries aim to promote patient-centered, effective, efficient, and collaborative health systems. Efforts to reform health service delivery systems to develop a more integrated system have been observed in many countries, including China, Ethiopia, the Philippines, Nigeria, Cameroon, Indonesia, and Zambia^1–4^. China has established and piloted medical alliances in both urban and rural areas as one of its major health service forms^5–8^. We use the term medical alliance to refer to a group of health facilities that collaborate to strengthen health service delivery, regardless of whether they are located in urban or rural areas. This paper provides an overview of the design and operational features of the public medical alliances in China.

## Three-tier PHC delivery system in China

**Fig. 1:**
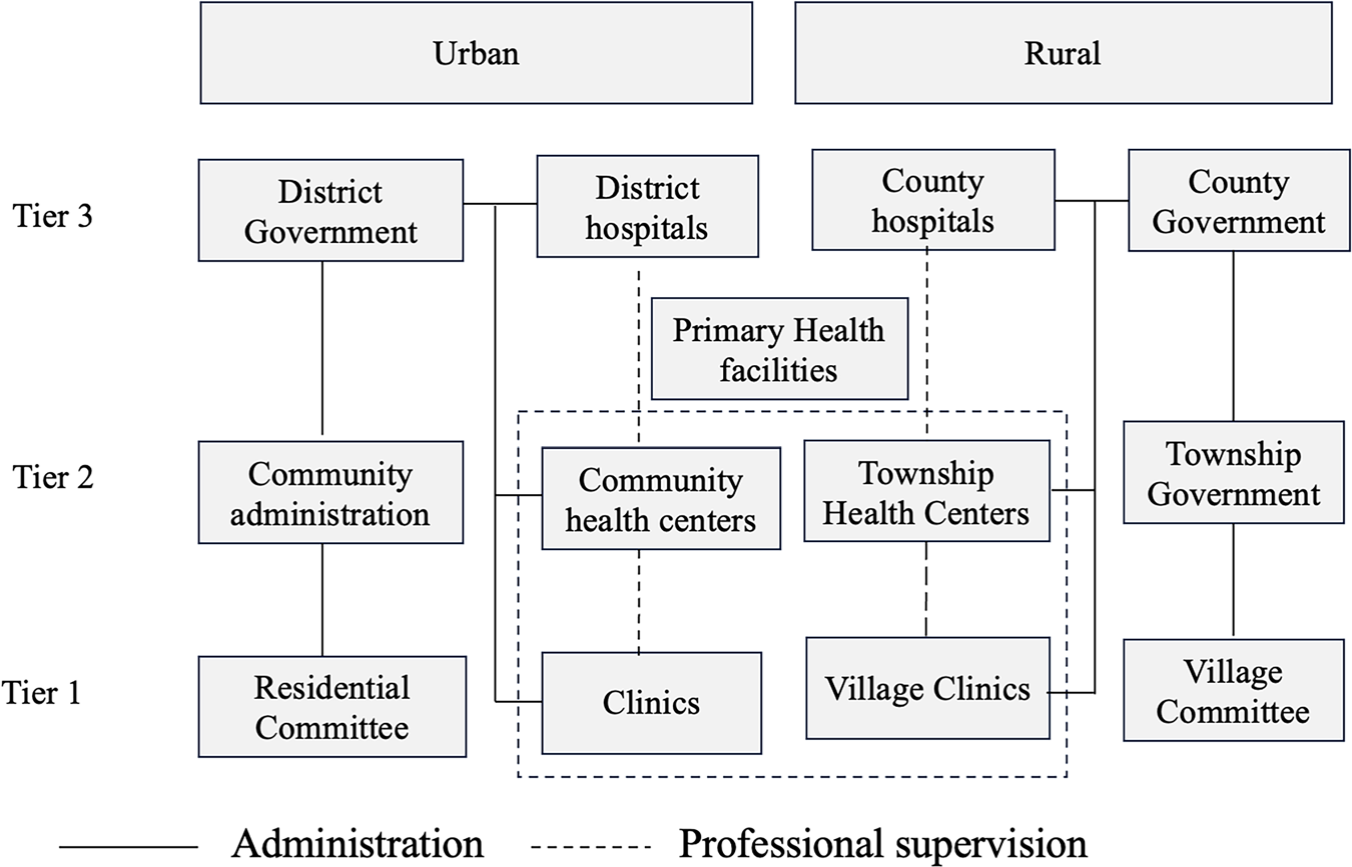
Three-tier PHC delivery system in China.

Source: Adapted from Feng et al.^9^ and Qin and Zhang^10^

Traced back to the 1950s, the three-tier primary health care (PHC) network was first established in rural China and included the brigade level composed of village clinics (VCs) attended by barefoot doctors, the commune level consisting of township health centers (THCs), and the county level of county hospitals (CHs)^9^. Accordingly, in urban areas, the three-tier network consisted of community health stations (CHS), community health centers (CHCs), and district hospitals (DHs) (Fig. 1). There were city or specialized hospitals that offered tertiary care or specialized care, respectively.

District and county governments, through their health bureaus, provide administrative leadership of the three-tier system in the urban and rural areas, respectively, while health facilities in different tiers have more professional rather than administrative relationships. After 1978, while the three-tier system was never formally abolished, many of its core PHC-centered and cooperative frameworks dissolved. The healthcare delivery system in China was criticized for being highly fragmented and poor coordination in providing health services to the community^11^.

## Forms of medical alliances

To improve health service delivery, China has implemented a series of reforms^12^. In 2016, China laid out its “Healthy China 2030,” painting the vision of China’s future health system: Universal Health Coverage with a patient-centered integrated care system^8^. To implement it, China has established and promoted medical alliances in both urban and rural areas. As of 2019, 1408 urban medical alliances in 118 cities and 3346 county medical alliances (CMAs) in 567 counties had been established, accounting for approximately 40% of the total number of cities and counties in China^13^. While the degree of integration of PHC into higher-level services varies, the common structures include:

(1) mergers of hospitals and PHC facilities, which involve major vertical structural changes across all participating entities (fully integrated model);

(2) outsourcing management duties of PHC facilities to hospitals (outsourcing model), where PHC facilities relinquish partial and full management authority and leverage the stronger management capacity of hospitals to enhance their clinical capacities while maintaining their financial independence; and

(3) providing technical support from hospitals to PHC healthcare facilities, with minimal structural changes to the participating entities (technical assistance model)^14^.

**Fig. 2:**
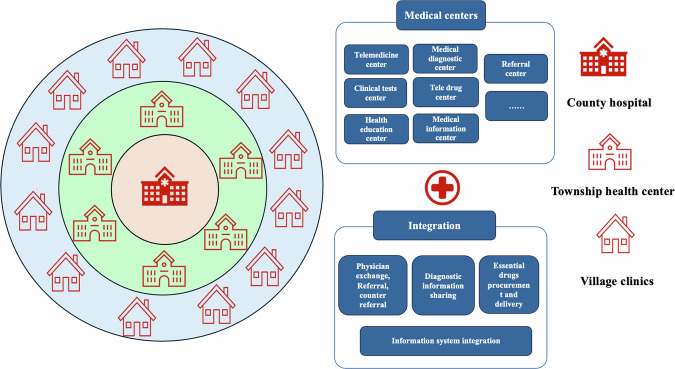
Ideal integration model of county medical alliances.

Figure 2 shows a model for county medical alliances, under which the leading CH takes the lead in consolidating resources in the area to restructure its medical units and uses various integration approaches to strengthen the capacity of THCs and VCs.

## Design and operational features of medical alliances

### Structure

Medical alliances are established based on zones (grids). A city or county is often divided into many zones (grids). One medical alliance in a grid is expected to consolidate and optimize health resources^15^. Structurally, a medical alliance in urban cities usually consists of one leading hospital (e.g., district hospital) and one or more PHC facilities (e.g., CHCs and associated CHS) in the same grid as the hospital. In this “1 + X” model, the leading hospital serves as the anchor of the alliance^16,17^. Occasionally, an alliance includes a tertiary hospital to strengthen the linkage between secondary and tertiary care and leverage the management and clinical capacity of tertiary hospitals to support the lower level of care^18^.

In rural areas, the common model of a medical alliance is “1 + X + Y”, consisting of one CH, several THCs, and associated VCs. The leading hospital (e.g., CH) collaborates with PHC facilities to provide accessible and quality healthcare services in rural areas. Multiple medical alliances can likely be established in a county^19^. With various structures of medical alliances, the internal relationship among participating health facilities (entities) within a medical alliance varies. Some alliances have to overhaul participating entities while others do not, with minimal changes. The government is poised to establish more fully integrated medical alliances.

To ensure the integration of healthcare, it is common practice to establish new units to manage the alliance and share the resources across participating organizations, depending on the need for medical alliances and the level of integration. The structural changes may include the establishment of clinical-related units and administrative units. In the fully integrated model, more centers are typically established, including information-sharing and service coordination platforms^14,17,20^.

### Governance

The establishment of a medical alliance is a government-led effort. Local governments (e.g., cities, districts, or counties) take responsibility for initiating the reform process with a task force to lead the reform^21^. The task force develops an overall strategic direction for integrated care reform.

The governance within a medical alliance takes various forms. Most fully integrated medical alliances are treated as a single juridical entity, with the participating institutions maintaining a certain level of autonomy^22^. However, medical alliances established using the outsourcing and technical assistant models are often not single juridical entities. Instead, leading hospitals and PHC facilities establish contracts to define their roles and responsibilities, with each participating entity having its juridical status^22^. An administrative council, generally consisting of directors of participating organizations with or without government officials, is established to lay out the mission, develop strategies, and make plans for a medical alliance, while the director of the medical alliance, who is often the director of leading hospitals, is tasked with managing daily activities within the medical alliance^23^.

The authority given to the director of a medical alliance is crucial and determines the degree of integration in a medical alliance. In the fully integrated model, the director of the medical alliance had a high degree of autonomy in managing resources (financial, human, and capital resources) within the medical alliance^14^. Under this model, the integration takes place in all aspects, including human resources, financing, procurement of medicines, information systems, and structure of service delivery. In contrast, under the technical assistance model, the director lacks such authority. Each institution within the medical alliance has its own governing body^22^. The collaboration among entities within the medical alliance is specified in a contract, which serves as the foundation for the collaboration. The scope of the contract varies along with the financial obligations, from human resource exchanges to diagnostic information sharing to service referral or counter referral.

To hold the director accountable for the performance of medical alliances, strong monitoring and evaluation (M&E) mechanisms are necessary. In 2018, the National Health Commission (NHC) released the performance evaluation guidelines for medical alliances^24^. Local medical alliances are encouraged to adapt the guidelines to their local contexts and develop more detailed performance assessment indicators. These specific performance indicators are designed to ensure service integration. Additionally, the government or health insurance funds are encouraged to tie the funding allocation to medical alliances to their performance on these indicators^24^.

Besides the performance indicators for medical alliances, specific performance indicators are designed for the directors of medical alliances and participating entities^24^. To ensure that the leadership of participating entities is motivated for integrated care, specific indicators that capture both clinical and financial measures of the entities are designed. So are the indicators measuring the integration of the care. The salary of the directors is tied to these indicators in some medical allances^21,25^.

### Financing and incentives

Financing and incentives are drivers of medical alliances toward patient-centered and integrated care. While fee-for-service remains the dominant payment method in most hospitals, China has made great strides in expanding health insurance coverage and reforming its payment system^12,26^. For example, the government finances the public health package on a per capita basis for essential preventive care and chronic disease management, and China is scaling up the global budget payment mechanism based on diagnostic-related groups (DRGs) in hospitals^23^.

With the ongoing provider payment reform, the prospective “global budget, profit retention” payment has been advocated for financially integrated medical alliances^14,22^. Under this policy, a global budget is estimated based on the combined payments to respective entities previously within the alliance, adjusting for inflation. This bundled payment to the medical alliance combines payments to hospitals and PHC facilities^22^. Once the medical alliance receives payments, it then allocates payments to participating entities based on contractual agreements. To buffer the potential financial risk, some funding is set aside as a risk adjustment fund^27^. However, for medical alliances that are not financially integrated, no payment alternation is performed, and they often keep their original payment mechanisms from various funding sources (e.g., government budget and health insurance funds).

To ensure that PHC facilities are better off, the structure changes under the medical alliance prioritize PHC facilities’ financial interests, and the government stipulates that the payments to PHC health facilities should not be lower than what was paid before the reform. Additionally, there have been policy changes to allow PHC facilities to use profits as bonuses to their health personnel^22^. The profit generated by medical alliances will be distributed to all participating entities according to a pre-determined formula, allowing PHC facilities to benefit from the good performance of the medical alliance^28^. The profit distribution financially ties PHC facilities and leading hospitals together to promote efficiency for better service delivery^28^.

Additionally, pay-for-performance (P4P) is encouraged to be used in medical alliances^24^. P4P has been implemented even before the establishment of medical alliances to reward good performance^29,30^. To accommodate the structure change in medical alliances, performance measures are redesigned or developed to evaluate participating organizations, directors, and individual staff^24^. National Health Commission advocated to maximize the use of performance measures, including the payment and the accreditation of medical alliances^24^. Some measures are designed to encourage specific behaviors. For example, to encourage counter-referral to PHC facilities, an indicator measuring counter-referral was used to evaluate the performance of physicians in the leading hospital^24^.

To encourage family medical teams to contract with individual households, thereby making PHC facilities the first point of contact for health services, a signing bonus is given to the team^14^. Furthermore, specific health insurance features are designed to encourage the population to use PHC facilities as the first contact, such as lower deductibles for inpatients referred by family doctors^31^. These incentive features reinforce patients’ and health workers’ behavior toward more coordinated care with a better PHC focus.

### Integration of health services

The integration of healthcare is based on the division of labor between PHC facilities and hospitals. In a medical alliance, the leading hospital is responsible for treating serious illnesses and providing technical support to PHC facilities, while PHC facilities tackle minor and common diseases. Information sharing is the core of service integration between hospitals and PHC facilities. To promote efficiency, patients’ medical records, diagnoses, testing results, and treatment regimens are shared within the medical alliance to facilitate referral and counter-referral^20^. This is possible due to the Chinese government’s investment in building a unified information infrastructure in both hospitals and PHC facilities^32,33^. Meanwhile, medical alliances establish centralized lab testing and imaging units for all patients in the medical alliance. The results from the shared centers are accessible by participating health facilities when needed by them.

The integration of services within a medical alliance is manifested by establishing a central medicine procurement and distribution system to address medicine shortages in PHC facilities. To improve medication accessibility in PHC facilities, several interventions have been observed within medical alliances^14,22^. First, a standard list of medicines is developed, which means all participating health facilities, whether they are PHC facilities or hospitals, would have the same catalog of medicines in stock. Second, the central procurement system, capitalizing on the stronger purchasing capacity of hospitals and their significant market influence, aims to procure medicines at potentially lower prices from pharmaceutical suppliers^34^. Third, these procured medicines are centrally stocked and distributed based on the anticipated needs of the participating organizations. These interventions substantially improve the affordability and accessibility of medicine in medical alliances.

Service integration is also reflected by the human resource exchange aimed at improving the quality of care in PHC facilities. PHC facilities have faced increasing criticism for the limited training of health personnel. To address this concern, hospital doctors are encouraged to provide health services in PHC facilities regularly, through which clinicians at PHC facilities learn from the practice^19^. Giving lectures and providing training to PHC clinicians are also encouraged. Alternatively, PHC clinicians can be offered a position to receive clinical training in the leading hospital within the medical alliance at no cost. This two-way exchange of human resources helps improve the clinical capacity of health staff in PHC facilities.

The integration of service within a PHC facility is enhanced by establishing family doctor teams to provide continuous and individualized care. Since 2016, registering individual households with family doctor teams that provide preventive care, health record keeping, telemedicine, and integrated elderly care has been launched and advocated^35^. A family doctor team generally consists of a general practitioner, a nurse, a public health physician, and a pharmacist, with the potential addition of other professionals as needed. The family doctor teams serve as gatekeepers for patients and provide more individualized care. Such practice improves patients’ willingness to use PHC as the first contact^35^.

## A snapshot of performance of medical alliances

Overall, integration has been prevalent in medical alliances. According to the National Health Commission, as of 2021, 93% of pilot counties had set up administrative councils for medical alliances, 72% had managed drug supply centrally, 76% had shared information within the alliances, 87% had developed referral and counter-referral guidelines, and 65% had integrated financial management^36^. It was reported that the share of outpatients in piloted countries with medical alliances rose to 55% in 2020, 2.3% higher than that in 2019, and 77% of patients with chronic conditions sought care in PHC facilities in 2020, 2.2% higher than that in 2019^36^. Studies at the subnational level also showed that medical alliance was associated with the increase of efficiency in county hospitals^37^, improved continuity of health care^38^, increased use of primary care and reduction of tertiary care^20,38^, and reduced health expenditure or reduced growth rate of health expenditure^20,39^.

## Potential issues of medical alliances

Despite its promise, fully realizing dual referral may be a lengthy and challenging process, especially in terms of counter-referral. Hospital doctors are often reluctant to refer patients back to PHC facilities due to several reasons. First, most hospitals are still under FFS, particularly those in the medical alliance using the technical assistance model, and they have no financial incentive to send patients back to PHC facilities to retain the patients at the hospital to generate revenue^40^. Second, there is still a large population that tends to seek care directly from hospitals. The government allows the public to visit hospitals for any disease and has no strict policies on gatekeepers. As a result, patients opt for hospitals over PHC facilities due to perceived differences in quality of care. Counter-referral is thus not favorable by patients. Third, economic incentives for the hospitals to receive referred patients are inadequate. Under the global budget framework, an increase in annually referred patients to hospitals does not necessarily correlate with an increase in funding/budget to those same hospitals. To preserve revenue, hospitals have little motivation to refer patients back to PHC facilities^41^. Fourth, health facilities sometimes lack clear standards for referral and counter-referral, resulting in patient referrals being largely dependent on the discretion of physicians. Thus, to mitigate medical risks associated with counter-referral to PHC facilities, hospital physicians may not choose to pursue this option^42^.

## Conclusion

Establishing an effective medical alliance entails complementary elements in a health system and beyond. Firstly, it requires strong political commitment. Reallocating the government’s health budget to establish medical alliances is not merely a technical issue but primarily a political one, necessitating paramount political commitment from the top leadership. Secondly, improving health financing is critical in triggering health service delivery reforms. Health investment empowers the government to take initiatives (e.g., restructuring its delivery system and developing payment mechanisms) to facilitate the establishment of a medical alliance. Thirdly, designing incentives, such as linking payment to performance, to reinforce desired behavior, is a crucial element in ensuring medical alliances meet their objectives. Fourthly, M&E serves as a mechanism to ensure the continuous improvement of the medical alliance’s functionality, such as identifying gaps, proposing appropriate actions, and implementing actions to address the gaps. It should be noted that the evidence of the impact of medical alliances in China on health service delivery remains limited. Research should focus on generating evidence on the successful operations of medical alliances and understanding how the local context affects their implementation.

## Data Availability

No datasets were generated or analysed during the current study.
